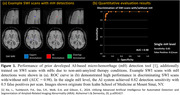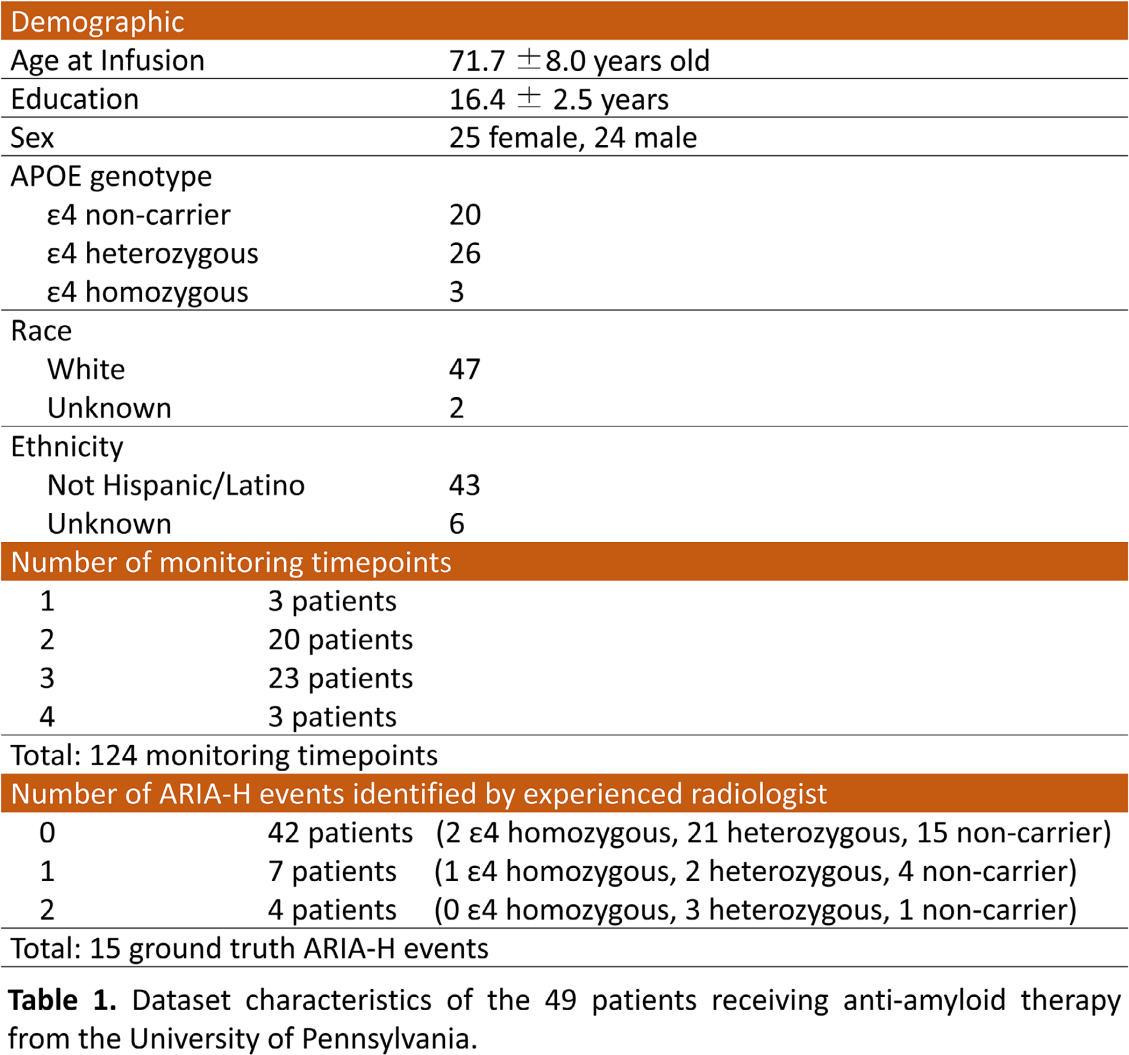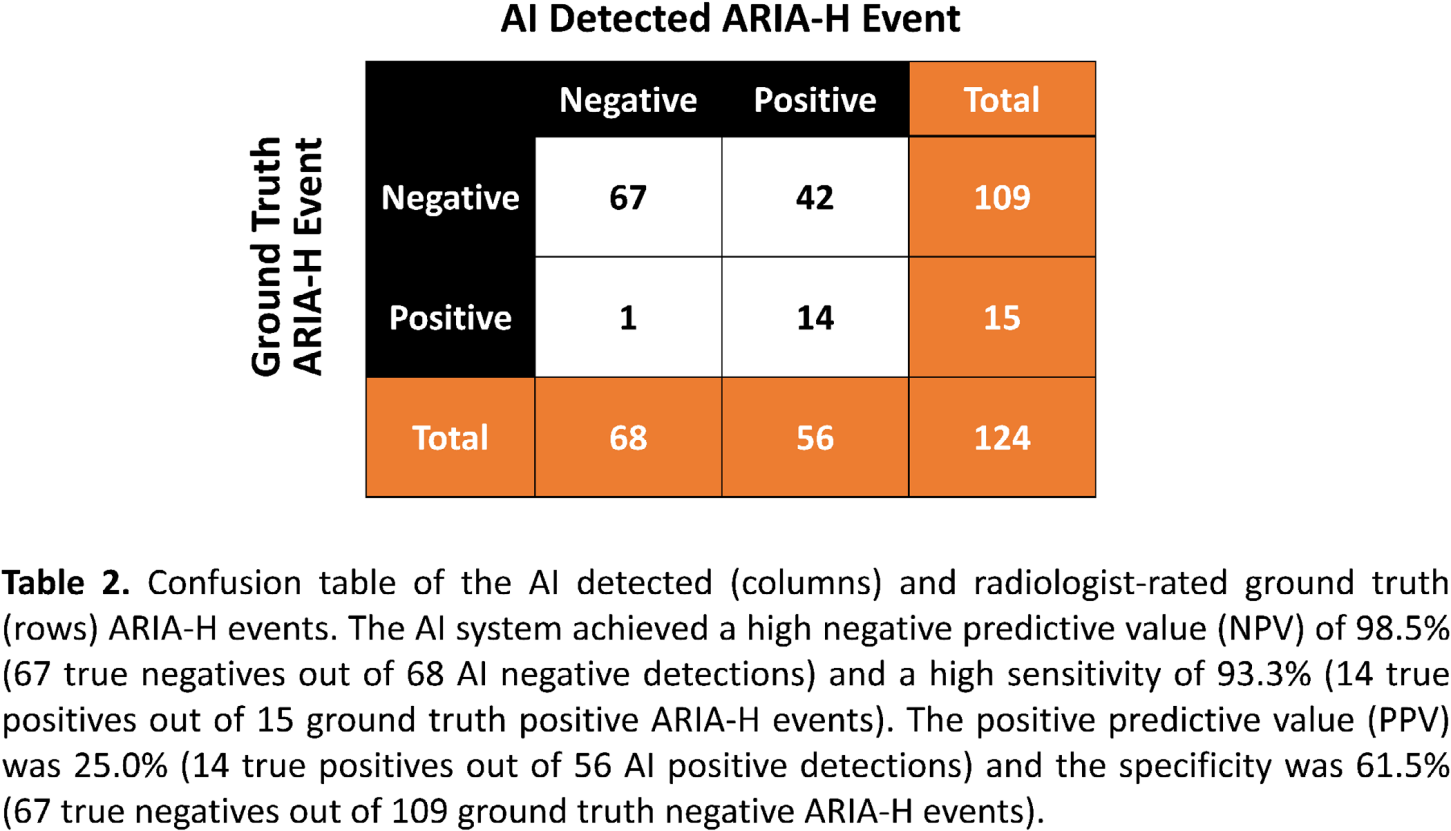# Artificial Intelligence Monitoring of ARIA‐H in Real‐World Patients Receiving Anti‐Amyloid Therapy

**DOI:** 10.1002/alz70861_108066

**Published:** 2025-12-23

**Authors:** Long Xie, Christopher A Brown, Ilya M Nasrallah, Manuel Taso, John A. Detre, Eli Gibson, Paul A. Yushkevich, Sandhitsu R. Das, David A. Wolk

**Affiliations:** ^1^ Siemens Healthineers, Princeton, NJ USA; ^2^ University of Pennsylvania, Philadelphia, PA USA; ^3^ Siemens Medical Solutions, Malvern, PA USA; ^4^ Siemens Heathineers, Princeton, NJ USA

## Abstract

**Background:**

Effective monitoring of amyloid‐related imaging abnormalities (ARIA) is a safety requirement with anti‐amyloid treatments for Alzheimer’s disease (AD). However, variability in clinical settings, limited availability of expert neuroradiologists, and the substantial volume of medical images required for ARIA monitoring pose significant challenges, reducing accessibility and increasing healthcare costs. Our previously developed artificial intelligence (AI)‐based ARIA detection tool (Figure 1) has the potential to address these challenges but has not been evaluated with “real‐world” AD patient data. This study aims to assess the effectiveness of our ARIA tool in detecting ARIA‐H events in patients undergoing anti‐amyloid therapy.

**Methods:**

We included longitudinal susceptibility‐weighted‐imaging (SWI) MRI scans of 49 patients receiving anti‐amyloid therapy at the University of Pennsylvania (Table 1). Experienced radiologist identified ARIA‐H events from the 124 monitoring timepoints by detecting new micro‐hemorrhages or superficial siderosis, serving as the ground truth. Our AI tool, based on a foundation model additionally trained on SWI scans with micro‐hemorrhages due to non‐anti‐amyloid therapy conditions, was applied to each SWI scan to count micro‐hemorrhages. If a monitoring timepoint showed more micro‐hemorrhages than the previous timepoint or the baseline timepoint, it was considered a positive AI‐detected ARIA‐H event. The AI system’s performance was measured by comparing the AI detections to the radiologist‐rated ground truth using negative predictive value (NPV), positive predictive value (PPV), sensitivity and specificity.

**Results:**

22.4% of patients (11 out of 49) developed ARIA‐H, consistent with the ARIA‐H rate observed in clinical trials. As shown in the confusion table (Table 2), out of 68 negative AI‐detected ARIA‐H events, 67 were true negatives, resulting in a high NPV of 98.5%. In addition, the AI system achieved 93.3% sensitivity in detecting ground truth positive ARIA‐H events (14 out of 15). The PPV and specificity were 25% and 61.5% respectively.

**Conclusions:**

The findings demonstrate that the proposed AI system can be used to identify low‐risk monitoring timepoints for ARIA‐H. This suggests potential for reducing radiology workload and serving as a screener for radiology evaluation, thereby enhancing the efficiency, cost‐effectiveness, consistency, and accessibility of ARIA‐H monitoring. Future work will incorporate longitudinal ARIA‐H training data to improve the PPV and specificity.